# Designing an Adaptive Adolescent Physical Activity and Nutrition Intervention for COVID-19–Related Health Challenges: Formative Research Study

**DOI:** 10.2196/33322

**Published:** 2022-01-21

**Authors:** Amanda Grimes, Joseph S Lightner, Kimberly Pina, Evelyn S Donis de Miranda, Emily Meissen-Sebelius, Robin P Shook, Emily A Hurley

**Affiliations:** 1 School of Nursing and Health Studies University of Missouri-Kansas City Kansas City, MO United States; 2 Division of Health Services and Outcomes Research Children’s Mercy Kansas City Kansas City, MO United States; 3 Center for Children’s Healthy Lifestyles & Nutrition Children’s Mercy Kansas City Kansas City, MO United States; 4 Department of Pediatrics University of Missouri-Kansas City School of Medicine Kansas City, MO United States; 5 Department of Population Health University of Kansas Medical Center Kansas City, KS United States

**Keywords:** intervention, physical activity, nutrition, adolescents, formative research, COVID-19, exercise, young adult, teenager, focus group, qualitative, interview, urban, barrier

## Abstract

**Background:**

With rates of childhood obesity continually increasing, effective physical activity and nutrition interventions are needed. Formative research is used to tailor interventions to different cultural and geographic contexts and can be vital in adapting intervention strategies in the face of significant disruptive circumstances (like COVID-19).

**Objective:**

We conducted formative research via in-person and web-based focus groups among middle schoolers and parents to better understand the facilitators and barriers to physical activity and fruit and vegetable consumption and to inform the design of a large intervention for a low-income, urban setting in the US Midwest.

**Methods:**

We conducted 2 phases of qualitative focus groups with parents (n=20) and 6th-9th grade middle schoolers (n=23). Phase 1 was conducted prior to the COVID-19 pandemic in late 2019, and phase 2 was conducted during the COVID-19 pandemic in the summer of 2020. Focus groups were transcribed and thematically coded using the Dedoose software.

**Results:**

The main facilitators of physical activity prior to the pandemic included the opportunity to have fun, peer influence, competition (for some), and incentives, while the main barriers to physical activity were time constraints and social discomfort. The main facilitators of eating fruits and vegetables included parental influence, preparation technique, and convenience, while barriers included dislike of vegetables, time constraints, and preparation or freshness. During the pandemic, facilitators of physical activity remained the same, while additional barriers to physical activity such as lack of motivation and limited time spent outside of the home were reported. For fruit and vegetable consumption, both facilitators and barriers remained the same for both time periods. Additionally, for some participants, the pandemic offered an opportunity to offer more fruits and vegetables to middle schoolers throughout the day.

**Conclusions:**

Some themes identified were common to those reported in previous studies, such as peer influence on physical activity and parental influence on fruit and vegetable consumption. Novel themes such as lack of motivation to be active and limited time outside the home helped improve intervention adaptation, specifically during the COVID-19 pandemic. The continuity of formative research after a major unexpected change in the intervention context can be essential in targeting areas of an intervention that can be retained and those that need to be adjusted.

## Introduction

Inactivity, poor nutrition, and obesity are pervasive and growing problems among children in the United States. In 2019, 71.3% of middle schoolers did not meet the recommendations of 60 minutes of moderate to vigorous physical activity daily—an increase from 67.6% in 2011 [1]. Fruit and vegetable consumption follow similar trends as physical activity. Among adolescents in 9th-12th grade, 41.8% consumed less than one fruit and 40.7% less than one vegetable per day in 2019, compared to 38.7% (<1 fruit) and 35.8% (<1 vegetable) in 2001 [2]. These deteriorating rates of physical activity and fruit and vegetable consumption are reflected in the growth of childhood obesity, which has increased from 14.8% in 1999 to 21.2% in 2018 for 12-19-year-old adolescents [3]. Further, racial minority and socioeconomically disadvantaged youth are at an increased risk to be overweight or obese [4]. Although evidence is limited and lacking for middle schoolers, recent research has indicated that the COVID-19 pandemic has negatively impacted physical activity and nutrition for other populations [5-7], indicating that it has also likely exacerbated negative physical activity and fruit and vegetable consumption trends seen among middle schoolers. In response, the US Department of Health and Human Services established the Youth Engagement in Sports: Collaboration to Improve Adolescent Physical Activity and Nutrition (YES Initiative), specifically aiming to increase physical activity and consumption of fruits and vegetables [8].

Physical literacy and fitness, motivation, positive attitude, as well as friends and family were all found to be dominant facilitators of physical activity for youth [9-13]. The nature of the activity has also been identified as a facilitator of physical activity; for example, being fun was the primary feature, while competition appears to be a facilitator for youth who are already active [10,14]. Similarly, numerous barriers for youth to being more physically active are supported by the literature. Common barriers include availability or access to physical activity programs [9,14]; past negative experiences associated with physical activity; appropriate resources available; inactive, inappropriate level, or nonmeaningful sessions; and nonsupportive environments, which were just some notable barriers identified in a review of the literature [10]. Lacking the necessary skills for youth’s preferred activity was also identified as a significant barrier [13]. Body image or gender-based sociocultural norms were identified as specific barriers for adolescent females [10]. Despite the breadth of research on this topic, it is important to tailor interventions to youth’s characteristics, interests, and circumstances [9,10].

Previous research has identified a number of facilitators of fruit and vegetable consumption for youth. A review by Patrick and Nicklas [15] found that both family and availability are strong facilitators of fruit and vegetable consumption. Other research identified self-efficacy and benefits were predictors of fruit and vegetable consumption for a sample of minority high school students, whereas social support was a predictor for nonminority students [16]. Lucan et al [17] found that health, taste, and preferences were the top facilitators of fruit and vegetable consumption. In a nationally representative sample of teenagers, it was found that normative belief was a strong predictor for male fruit and vegetable consumption, while perceived barriers were strong predictors of female fruit and vegetable consumption [18]. Costs, availability, and preferences rank as the top barriers to fruit and vegetable consumption [17].

Interventions to increase physical activity may be particularly important in the middle school years. For example, only 7.9% of middle schools in the United States provide daily physical education for the entire school year [19], indicating a need for additional physical activity programming for this age group. Participation in sports may be an effective strategy to help youth meet the current physical activity guidelines [20,21]. Since sport participation disparities exist by race, socioeconomic status, and gender [22,23], it is important that interventions address barriers to participation specific to these at-risk populations.

A critical component of developing an effective intervention is formative research, which aims to identify how to best implement aspects of a program to the context of its stakeholders [24]. Formative research is an iterative process to ensure that components of an intervention are informed by the direct experiences and opinions of the local population it intends to benefit [24]. Previous research suggests that a 2-phase formative research process is ideal to satisfy the immediate need of the information to proceed with intervention development and implementation as well as providing an opportunity to gain follow-up as additional needs and challenges are identified [25]. In our case, these phases were separated by the onset of the COVID-19 pandemic, presenting even more challenges in implementing an intervention originally designed for the in-person school setting.

Research on the barriers to and facilitators for physical activity and fruit and vegetable consumption specific to middle schoolers is lacking; this gap in literature is particularly important because this is a pivotal time for developing health habits. Therefore, the overall objective of this paper is to describe how formative research informed a large-scale intervention that meets the goals of the YES Initiative for middle schoolers prior to and amid a global pandemic. More specifically, we aim to describe formative research findings from a 2-phase process, their impact on intervention implementation, and how both formative research and intervention implementation pivoted due to COVID-19–related challenges.

## Methods

### Design

We conducted a series of qualitative focus groups, separately with middle school students and their parents, in 2 phases (before and after the onset of COVID-19). Compared to surveys, focus groups allow for rich, open-ended responses about participants’ ideas and experiences that we needed to help shape intervention components in the early stages of development. Compared to individual interviews, focus groups served as an efficient way to gather perspectives of a large sample and offered the advantage of interaction among participants, which can help illuminate areas of common experience and formulate consensus around programmatic recommendations [26]. Further, segmentation of each study population (parents and students) may increase the openness, comfort level, and ability of participants to relate to each other’s experiences, allowing for richer responses than if groups were mixed [27].

Both phases of focus groups aimed to gain a deeper understanding of the following topics: (1) facilitators and barriers to physical activity, (2) parent/student recommendations for increasing physical activity through sports/activities, (3) facilitators and barriers to fruit and vegetable consumption, and (4) parent/student recommendations for increasing fruit and vegetable consumption through a fresh produce and nutrition education intervention. Focus group guides were informed by the social-ecological model [28] and social cognitive theory [29] (Multimedia Appendix 1). The social-ecological model offers a multi-level perspective to examine factors influencing behavior at the individual and environmental (family, school, and community) levels. Embedding selected elements of social cognitive theory within our framework provided further direction on specific influences to examine at the individual level (self-efficacy, interests, motivations, experiences) and environmental (social support, opportunity) levels. Further, both theories have been used in combination to inform past formative research and successful interventions in healthy lifestyles among adolescents [25].

### Recruitment

All procedures were approved by the Institutional Review Board at the University of Missouri-Kansas City prior to study commencement. Initial procedures were updated to reflect protocol changes necessary to accommodate COVID-19 risk mitigation (ie, virtual focus groups via Zoom); updated procedures were approved prior to commencement of phase 2 focus groups. Phase 1 focus groups were conducted in-person in December 2019, and phase 2 focus groups were conducted virtually in the summer of 2020 (June-August). Participants in each phase were unique.

Participants in the first phase were recruited prior to implementing the intervention through the local children’s hospital’s daily newsletter and represented 8 school districts in the metropolitan area. A total of 4 focus groups were conducted in this phase in December 2019—2 with parents (n=5, n=6) and 2 with middle schoolers (n=6, n=6).

The second phase of focus groups was conducted in June-August 2020, after participating schools were closed due to COVID-19 and the intervention was suspended. Participants in this phase were recruited from middle schoolers enrolled in control and intervention groups and were invited to participate via email. Recruitment emails sent by research staff indicated that focus groups would take place virtually via zoom and that participants would need the necessary technology to participate. Four focus groups were conducted in this phase—2 with parents (n=2, n=3) and 2 with middle schoolers (n=4, n=6). In this phase, an additional Spanish-speaking parent focus group (n=4) was conducted in response to interest expressed by parents who had previously been unable to participate owing to language barriers. Participants in each phase, both parents and middle schoolers, received a US $30 gift card.

### Data Collection and Analysis

All focus groups were conducted by members of the research team trained in focus group moderation for formative research (EAH, KAP, ESDM, EMMS). For in-person focus groups in phase 1, participants gave informed consent and completed a demographic survey on paper prior to the start of the discussion.

Virtual focus groups in phase 2 were designed to assess the transferability of findings identified in the first phase of focus groups to the changing intervention context presented by COVID-19. Themes that were approaching saturation from phase 1 were included in the guide for phase 2 for the purposes of confirming initial findings but not to explore in great depth (eg, influence of friends). New subtopics that emerged in phase 1 and did not yet reach saturation were added to the guides for the second phase for exploration in greater depth, specifically issues surrounding body image and use of recipes. Phase 2 also included new topics related to COVID-19, specifically (1) the pandemic’s impact on physical activity and fruit and vegetable consumption and (2) recommendations for web-based program adaptation.

Participants in phase 2’s virtual focus group gave informed consent over the phone and completed a demographic survey online prior to the scheduled discussion session. During the discussion, participants had the option of using video or audio only and could use the chatbox. Recordings were made only of audio and chatbox content. For Spanish groups, all consent forms and focus group guides were translated by bilingual members of the research team (KAP and ESDM) according to a 9-step process [30,31].

Verbatim transcripts were produced for English-speaking focus groups using the automatic transcription feature in Zoom and then manually improved for accuracy alongside the audio-recording. For Spanish-speaking groups, verbatim transcripts were produced manually by the bilingual research team members and then translated into English. Transcripts were uploaded to the Dedoose app for analysis [32]. An initial coding tree was developed based on the starting theoretical frameworks (social-ecological model, social cognitive theory) and discussion guide topics. Three coders coded the same first 4 transcripts from phase 1 independently, using a combination of deductive coding based on the initial coding tree, while also allowing for adjustment and addition of the thematic subcodes inductively [33]. The coding team met to debrief and synthesize codebook revisions, resolve discrepancies (with the senior author serving as the final arbitrator), and synthesized a final codebook for the remaining phase 1 transcripts, which were coded independently. The same codebook was applied to the phase 2 transcripts by independent coders, while adding new codes inductively as needed, which were integrated into the final codebook after group consensus. Summary outputs were examined by participant type and time of collection, discussed, and synthesized into themes. Throughout data collection and analysis, the study team tracked thematic saturation through memoing and debriefing, determining saturation through consensus.

## Results

### Demographics of the Participants

Table 1 outlines participants’ demographics. The mean parent participant age was 41 years, and the mean middle schooler participant age was 13 years. Participants (parents and middle schoolers) predominantly spoke English (16/20, 80% and 23/23, 100%; respectively). The majority of parents were females (19/20, 95%), but the majority of middle schoolers were males (13/23, 57%). Parents and middle schoolers were predominantly non-Hispanic (15/20, 75% and 16/23, 70%; respectively) and White/Caucasian (14/20, 70% and 12/23, 52%; respectively).

**Table 1:** Demographic characteristics of the focus group participants.

Characteristics			Parents (n=20)		Middle schoolers (n=23)
**Language, n (%)**					
	English	16 (80)		23 (100)	
	Spanish	4 (20)		0	
Average age, median (IQR)			41 (31-64)		13 (11-15)
**Gender, n (%)**					
	Male	1 (5)		13 (57)	
	Female	19 (95)		9 (39)	
	Prefer not to answer	0 (0)		1 (4)	
**Race, n (%)**					
	White/Caucasian	14 (70)		12 (52)	
	Black or African American	2 (10)		5 (22)	
	Multiracial	0		1 (4)	
	Other	4 (4)		4 (18)	
	I prefer not to answer	0 (0)		1 (4)	
**Ethnicity, n (%)**					
	Non-Hispanic	15 (75)		16 (70)	
	Hispanic	4 (20)		5 (22)	
	I prefer not to answer	1 (5)		2 (8)	
**Focus group type, n (%)**					
	In-person	11 (55)		12 (52)	
	Virtual	9 (45)		11 (48)	
**Grade level, n (%)**					
	6th	N/A^a^		3 (13)	
	7th	N/A		8 (35)	
	8th	N/A		6 (26)	
	9th	N/A		6 (26)	

^a^N/A: not applicable.

### Physical Activity

The main facilitators of physical activity were similar before the pandemic and amid the pandemic and included having fun, peer influence, competition (for some), and incentives. The main barriers were time constraints, social discomfort, and additionally during the pandemic, limited time spent outside of home.

#### Facilitators

In phase 1, middle schoolers and parents cited peer influence and socialization as the strongest motivator for physical activity. Students enjoyed being active if it meant having fun with friends they were comfortable with, although they also noted they enjoyed meeting new people through physical activity.

…When we play with friends it makes it more fun. When you know the people that you're playing with or against.

Phase 1, male student

Some middle schoolers and parents noted students’ desire for competitive sports and games.

…I like to play to win and I just play to have fun, but I like to play to win.

Phase 1, male student

…He has this mindset of drive and focus and winning and competition. And I don't know what to do with it but let him play.

Phase 1, English-speaking female parent

In phase 2, peer and social influence were again noted as the strong motivators for physical activity. Similar to sentiments in phase 1, middle schoolers emphasized enhanced enjoyment of activities played with peers they considered friends.

…It’s more fun when you play against your friends because with strangers you don't feel as comfortable.

Phase 2, female student

Parents and students again emphasized the potential benefits of competition and incentives. Amid pandemic-related restrictions, some middle schoolers described how they continued to seek competitive environments, and parents suggested ways for the intervention to preserve the social aspect of competition as motivation for physical activity.

…I went against my friend to see who gets the most steps in a day.

Phase 2, male student

…Maybe given them an incentive, so now that they are still young get motivated to do some type of activity because now, many cannot get together. They could get together to know each other, if they were about the same age, do some type of challenge. But virtually, incentivize them so they want to participate virtually.

Phase 2, female parent

#### Barriers

In phase 1 focus groups, barriers to physical activity were often related to time constraints and restrictive social factors. For example, some students struggled with finding time to participate in physical activity while managing homework and other obligations. Although previously noted as a facilitator for many middle schoolers, competitive environments were noted by others as creating potential conflicts among peers.

…I play for my school volleyball team and there is drama between some people on the team. So that was kind of affecting some people and made it a little less enjoyable to play.

Phase 1, female student

Others expressed the fear of feeling “awkward” in competitive environments, with anxieties about not knowing how to play the sport, making an error, or not being as good at the sport as their peers.

…If you're not good at it, and maybe those people that you play against are good at it, then it's not as fun.

Phase 1, male student

In phase 2, participants described how COVID-19 decreased physical activity among middle schoolers overall.

…their physical activities [has] definitely slowed down. I mean, they still do go outside and stuff, but they don't play the way they used to.

Phase 2, female parent

Barriers to physical activity still included time and social constraints, and additionally, a decrease in motivation to remain active when in-person schools and activities were suspended.

…Like you don't feel like being like, like during quarantine or whatever you didn’t feel like going outside a lot, you just wanted to be inside a lot. You just felt like being inside like laying on your bed.

Phase 2, female student

Participants described additional COVID-19–related barriers, including the loss of opportunity for structured physical activity in the school environment and parental reluctance to let children venture far outside the home owing to the risk of contracting the virus.

…Yes, when they would come from school, they would also go outside with their bicycles. But now with the pandemic that they are here, with the school [online] they do not want to go out. They say that they have a lot of homework and they have who knows what. However, when she was going to school, they would give her an hour and all the stairs going up and down, all that helped a lot. Now, no matter how much I tell her [to be active] she doesn’t want to.

Phase 2, female parent

…What I've noticed is definitely a difference, since we've been more hibernated this year than normal like even if they go outside and stuff, but it's not the same as well. I mean, like, I wouldn't let them go to the park forever […] I have started at least letting them go sometimes because it's, you know, they, like I said, they love being outside, but it's just such a weird time.

Phase 2, female parent

#### Parent and Student Recommendations

As an extension of motivation by competition, parents and students suggested the benefit of incentives, challenges, or activity goals as intervention components, emphasizing the importance of connecting friends through these activities. Middle schoolers in phase 1 also said an ideal intervention would include a variety of participation options, including different sports and games and competitive and noncompetitive choices.

### Fruit and Vegetable Consumption

The main facilitators of eating fruits and vegetables included parent influence, preparation technique, and convenience, which were similar to that reported before the pandemic and amid the pandemic. The main barriers to eating fruits and vegetables included middle schoolers disliking vegetables, time constraints, and preparation or freshness.

#### Facilitators

Findings from phase 1 suggested that middle schoolers were aware that fruits and vegetables are good for their overall health. Parents reported needing to take a major role in encouraging their children to eat fruits and vegetables. They also noted that the cooking method or preparation technique (eg, cutting, peeling, adding dressing) determined if their child ultimately consumed them.

…I will puree anything that needs onions or tomatoes. If you get the immersion blender and make it almost like a sauce they never know. They will eat it.

Phase 1, female parent

…When it comes to fruits, honestly, I have to cut down the oranges, so they eat them, otherwise they don’t eat them. […] I need to push them so they eat the fruits and vegetables.

Phase 2, female parent

In phase 2, parents continued to be a primary influence on their children’s fruit and vegetable consumption. With school being held virtually and many parents working remotely, parents were able to offer vegetables conveniently and more frequently.

…One of the things that I've done during the quarantine […] I start out the day by cutting up fruits and vegetables and putting them out on the kitchen counter. So that they’re always available. So as my son walks through the kitchen like yesterday morning he comes down for breakfast, and he grabbed cucumbers off the counter.

Phase 2, female parent

Phase 2 also included additional discussion about recipes, which parents reported using regularly, appreciating those that made healthy meal preparation simple and convenient. English-speaking parents appeared to be more open to new recipes, while Spanish-speaking parents tended to seek out familiar recipes.

…As long as it's something simple and quick and easy and I don't have to get out and run to the store.

Phase 2, English-speaking female parent

…We actually do food prep on Sunday. My kids will help me on that. […] We like to try new recipes every time we cook. [We will] put them in the freezer so that when we head out, it's easy to just say, throw it in the oven and it'll be ready by the time we get home from any after school activity.

Phase 2, English-speaking female parent

…Sometimes, I use recipes from YouTube when I see them. Or sometimes I called my sisters to ask them how to cook something I want to make and they tell me how to make it.

Phase 2, Spanish-speaking female parent

#### Barriers

As a chief barrier in both phase 1 and 2, parents and middle schoolers discussed how children are often not fond of fruits and especially vegetables. When middle schoolers were asked about their frequency of fruit and vegetable consumption on a scale of 1 to 10, one student responded with the following quote regarding his fruit and vegetable consumption.

…Because I don't get any of the fruits and vegetables at school because they don't taste good and they make you get a fruit or vegetable. And an apple juice counts [as a fruit], so I just get an apple juice.

Phase 1, female student

Students also expressed their dislike for fruits and vegetables provided in school meals due to lack freshness, bad taste, and lack of variety.

…[Schools should] spend a little more money on making it more fresh, or buying them from better sources, I'd probably say. And just make them more local, and I think a lot more people want to eat fruits and vegetables at school if they tasted better. [Phase 1, male student)

Additionally, parents believed that constraints on their own time limited their ability to prepare healthy meals for their children. Further, parents identified time with their children’s grandparents as a negative influence, as they tended to be more permissive, allowing their children to eat unhealthy foods.

#### Parent and Student Recommendations

Parents and students emphasized that an intervention should promote healthy eating as a fun and engaging activity. Parents felt that if the children could prepare recipes themselves, that would give them a sense of pride and they would more likely eat the food. Students specifically talked about creating a cooking competition or a challenge where all students can be involved, as well as utilizing appetizing pictures and social media to engage students.

### Integrating Formative Research Findings Into Intervention Design

Findings from both phases of the focus groups helped to inform the intervention prior to and during the COVID-19 pandemic. The key themes and subsequent intervention strategies are summarized in Table 2. Phase 1 focus groups affirmed many proposed intervention strategies such as offering an intramural sports program (to create a motivating social environment) with rotating sport offerings (to ensure a variety of activities with competitive and noncompetitive options). We also aimed to achieve a positive social environment through positive sport coach training and referral incentives to increase friend participation. Gift cards incentives were also provided for meeting individual participation goals, facilitating motivation from goal setting without the pressure of peer competition for those who cited it as a barrier. Each youth was also provided a wearable, wrist-mounted accelerometer to measure physical activity and track steps, providing additional opportunity for goal setting and competition according to participant preferences.

By offering the program after school, we were able to reduce time constraint barriers with strategies such as utilizing an existing late-departing school bus. We also partnered with a local hospital’s “mobile market” bus to deliver fresh fruits and vegetables weekly, providing participants enough fruits and vegetables to make a meal for a family of 5 individuals. When COVID-19 interrupted the initial intervention plans, phase 2 focus groups helped determine how to adapt the intervention in light of the changing facilitators and barriers. Shortly after, we began offering live “Move Sessions” through Microsoft teams, allowing middle schoolers to engage with physical activity coaches and their peers. In response to parents’ mention of their child’s notable decline in physical activity during the pandemic, we expanded the Move Session offerings to 7 sessions per week, including multiple afternoon/evening options and 1 weekend option. We continued incentives for completing participation challenges. To increase exposure and variety, we provided all middle school participants with sports equipment (ie, jump rope, baseball and glove, yoga mat). Weekly produce distributions were maintained by making basic modifications (ie, no-contact drive-through, masks), and a weekly newsletter was developed to provide healthy recipes coordinated with the produce distribution.

**Table 2:** Key themes and subsequent intervention strategies.

Themes		Intervention strategies
**Physical activity**		
	Friend and peer influence	The intervention is free and open to all middle schoolers at a given school, with no try outs and regardless of level of ability. This easily allows for friends to participate (year 1 and 2).
	Competition or goal setting	We utilize activity trackers to provide opportunity for individual goal setting and personal competition (year 1 and 2). This sport sampling intervention allows for skill development and concludes with a scrimmage to allow for low-stakes competition between peers (year 1).
	Incentives	Middle schoolers can earn incentives (ie, gift cards) for reaching participation goals (year 1 and 2).
	Fun	Sport offerings rotate ever 2 weeks to keep the programming fun and new (year 1 and partially implemented in year 2). Provide all middle schoolers with sports equipment (ie, jump rope, baseball and glove, yoga mat) to increase exposure and variety (year 2).
	Time constraints	The intervention takes place at the middle schoolers’ respective schools that eliminates additional transportation needs and travel time and concludes prior to other potential evening activities (year 1). Move Session offerings were expanded to 7 virtual sessions per week, including multiple afternoon/evening options and 1 weekend option (year 2).
	Motivation	All coaches are trained in positive sport coaching to ensure a positive, inclusive, and motivating environment (year 1 and 2).
**Fruit and vegetable consumption**		
	Parental time constraint	Providing weekly produce packages has the potential to eliminate additional grocery trips (year 1 and 2).
	Disliking fruits and particularly vegetables	Each produce package contained common staple fruits and vegetables that tend to be well-liked as well as more unique or less accessible fruits to expand middle-schooler exposure.
	Recipes	Newsletters with recipes related to the produce selection are included with each produce bag (year 2).
	Preparation of fruits and vegetables	Newsletters that were included with each package of produce included preparation tips related to produce in the weekly package (year 2).

## Discussion

### Principal Findings

This formative research study informed the creation of a large-scale physical activity and nutrition intervention and its adaptations amid the onset of COVID-19. Overall, the main thematic findings from prepandemic focus groups were highly transferrable to the pandemic times, including primary motivators for participation in physical activity (peer influence, goal setting, or competition) and many barriers to consuming fruits and vegetables. However, later focus groups revealed that the pandemic introduced new facilitators (increased parental involvement in fruit and vegetable consumption) and challenges (declining motivation and limited structure for physical activity that needed to be incorporated into the revised intervention).

### Physical Activity

#### Facilitators

In both phases, we found that peer influence and socialization were the most commonly cited motivators for physical activity. These findings are consistent with previous findings that suggest that peer influence significantly impacts physical activity behavior over time in children [9-12]. Having fun also emerged as a dominant theme for physical activity participation, similar to that reported in other studies [10,13]. Despite the role peers play, our study found that parents had an increased role in facilitating physical activity during COVID-19, reflecting prior evidence that supports parental involvement can increase youth physical activity [9,10,29].

#### Barriers

Barriers to physical activity uncovered in our data were also similar to those reported in previous studies, such as time commitment (ie, homework obligations) [13,34], and for some, a counterproductive competitive environment for physical activity [10,13]. Our study further revealed that COVID-19 exacerbated these existing barriers and created additional barriers such as reduced physical activity and motivation to be active. These qualitative findings provide context for recent quantitative studies that confirm decreases in physical activity for children during the COVID-19 pandemic and more so for middle-school-aged children (age 9-13 years) compared to younger children (age 5-8 years) [35].

#### Parent and Student Recommendations

Students and parents suggested levering competition and goal setting as a strategy to increase physical activity. As such, incorporating wearable activity monitors that allow for self-tracking may provide an opportunity for both competition and goal setting. Previous research indicates that wearable activity monitors may be a particularly beneficial strategy to increase physical activity among students who are less active to begin with [36]. Lastly, participants also recommended the use of incentives to motivate middle schoolers. Although not widely studied in youth, evidence does suggest that providing financial incentives as done so in our intervention can increase physical activity among youth [37] but may be limited in sustaining long-term behavior change in sedentary adolescents [38].

### Fruit and Vegetable Consumption

#### Facilitators

Parents reported high levels of influence over their middle schooler’s food choices, controlling food availability and rules, as noted in previous studies [39,40]. This parental control often facilitated fruit and vegetable consumption; however, parents also mentioned struggling to get their child to eat vegetables, and they used presentation, preparation technique, and convenience to encourage healthy eating. The pandemic seemed to allow increased opportunity for parental control, as some parents mentioned offering more fruits and vegetables throughout the day. These findings align with other research that the pandemic potentially has had a positive effect on dietary behaviors in children [41].

#### Barriers

Middle schoolers reported liking very few vegetables, which aligns with that reported in previous research [40]. Yet, unlike that reported in other research, middle schoolers in this study reported knowing the benefits of eating healthy; therefore, it does not seem to be a significant barrier for this population [40]. Middle schoolers made specific complaints about the preparation and freshness of fruit and vegetable offered at schools, indicating that school-level changes may be needed to increase fruit and vegetable consumption. This may indicate that middle schoolers lack a supportive environment for fruit and vegetable consumption at school—an important factor in facilitating fruit and vegetable consumption [42].

#### Parent and Student Recommendations

Both parents and students alike suggested that a nutrition intervention should be a fun and engaging activity. One way to achieve this is through the promotion of cooking at home. In a review of the literature, researchers found that youth overall enjoyed cooking programs [43], and previous research suggests that cooking at home is associated with several nutritional benefits for youth [44]. Further, youth who are involved with cooking at home are more likely to enjoy cooking as an adult [45]. Parents also suggested novel approaches such as cooking challenges and using social media to engage students. There is currently a lack of research on these topics but they may have promise and should be explored.

### Strengths and Limitations

This study was strengthened by its qualitative methodology that allowed for deeper probing than alternative quantitative approaches. The inclusion of focus groups before and after a major interruption to intervention roll-out allowed for the adaptation of the intervention as well as a unique demonstration of the essential role of formative research to address unforeseen midprogram challenges. It also demonstrated the feasibility of shifting focus group data collection and intervention strategies to virtual platforms. This study was further strengthened by the inclusion of both English- and Spanish-speaking families.

As this was a formative research study intended to adapt an intervention to a specific context, results cannot be generalized from the limited sample size to the broader population of middle school students and parents. Sampling from phase 1 and phase 2 also differed demographically, as phase 1 participants were sampled at large and phase 2 directly from the schools involved in the intervention. Further, phase 2 focus groups were held virtually, meaning that participation and findings may have been different compared to an in-person setting (such as in phase 1). Although virtual focus groups allowed for participation among individuals for whom in-person sessions were not feasible, they required a conducive at-home environment (few distractions, good internet connection). Differing preference for use of chatbox and video may also have had different impacts on engagement and responses among participants.

### Conclusions

Our formative focus group discussions informed the creation of a large-scale physical activity and fruit and vegetable consumption intervention and its successful adaptation during the onset of COVID-19. These findings offer a feasible and targeted approach to increasing physical activity and fruit and vegetable consumption in middle school students through both a school-based in-person and virtual intervention. Moreover, this study demonstrates the importance of planned multi-phase formative data collection throughout a project. Future research must examine the efficacy of the intervention developed based on these findings.

## Appendix

Multimedia Appendix 1 Focus group discussion guides.

## Abbreviations

YES Initiative: Youth Engagement in Sports-Collaboration to Improve Adolescent Physical Activity and Nutrition
