# Supplementary material for: Designing an Adaptive Adolescent Physical Activity and Nutrition Intervention for COVID-19–Related Health Challenges: Formative Research Study
Source: JMIR Form Res. 2022 Jan 21;6(1):e33322. doi: 10.2196/33322 (PMC8785954; doi:10.2196/33322)
Supplement: Multimedia Appendix 1 [file formative_v6i1e33322_app1.docx]

**UMKC YES Initiative**

## Phase 1 Middle Schooler Focus Group Guide

**Aims:**

Identify the target population’s preferred produce that may include dark leafy greens, red and orange vegetables and methods to reduce consumption of sugar-sweetened beverages among target population. Identify barriers, concerns, and experiences accessing healthy foods.

Identify engaging sports activities for target population. Identify barriers, concerns, and experiences with performing activities.

**Time:** 60-90 minutes

**Supplies:**

- Audio recorders
- Favorite meal worksheets
- Notepad
- Easel notepads
- Markers
- Pencils
- Demographic survey
- Incentive
- Meal/Snacks
- Name tags

**Introduction:**

**Moderator**: Hi everyone, my name is Kimberly and this is *blank.* We are with Children’s Mercy and we work within the Health Services and Outcomes Research Division. We invited you here today asking for your help in starting a new before- or after- school program. We want to know your opinions and ideas about healthy eating in your family and your child playing sports.

The input you provide today will be used to help create this program and will remain confidential. We are audio recording the discussion today because we do not want to miss any of your comments. You all will say helpful things today and we cannot write fast enough to get them all down. Our reports from the discussion will go back to Children’s Mercy staff with no names attached and secured in our encrypted database.

Here is a document that talks about what it means to be in a study that we’ve given you. There is contact information for the study team as well as the hospital’s review board that oversees studies if you have any concerns or questions after today. Being in a study is completely voluntary, so you can choose to leave at any time or not answer any questions. By staying and participating in this discussion you are consenting to be in the study.

Do you have any questions about what it means to be in the study?

Let’s begin! Throughout the discussion today there will be a lot of ideas and some of you may be excited to talk about it. We encourage you to say anything that is on your mind. However, try not to interrupt someone when they are talking. You can speak after the person is done. If you have a cell phone, please put it on silent mode. If you need to go to the restroom, let us know.

*After everyone has arrived and filled out their favorite meal, have all the participants say their first name, what their favorite meal is and why they like it.*

**Topic 1: Fruits and Vegetables (20 min)**

|  | **Question** | **Goal** |
| --- | --- | --- |
|  | Icebreaker & Introductions: Favorite meal  *As participants arrive, they will be asked to fill out a worksheet where they can write down the recipe to their favorite meal. Once everyone is done, they will be asked to introduce themselves and share their favorite meal with others.* | Introductions, icebreaker |
| 1. | Thanks for sharing all the meals with us! Now I want to talk to us to talk some more about food, but more specifically fruits and vegetables. What are your thoughts about eating fruits and vegetables?  *Probes:*   - When I first said, “fruits and vegetables”, what was the first thought that came to your mind? - What fruits or vegetables do you like? What do you like about them? - Which do you not like? What is it that you don’t like about them? | Identify child-level attitudes about eating fruits and vegetables. |
| 2. | What does your family think of fruits or vegetables?  *Probes:*   - *How often do you eat fruits and vegetables at home?* - *What is it like for a family shopping in your community to get fruits and vegetables?* | Identify family influences on child fruits and vegetable consumption |
| 3. | What do other kids at school think about fruits and vegetables?  *Probe:*   - *How often do you eat fruits and vegetables with friends at school?* | Identify peer influences on child fruits and vegetable consumption |
| 4. | What makes it hard for kids your age to eat fruits or vegetables?  *Probes:*   - *What makes it hard to eat fruits and vegetables if they are right in front of you?* - *What makes it hard to eat fruits and vegetables to eat at school?* - *What makes it hard to eat fruits and vegetables with your family?* - *Do you have any worries about fruits and vegetables?* - *Can you explain a time where you had an obstacle or a barrier to eating fruits and vegetables?* | Identify barriers to eating fruits and vegetables at all levels |

**Moderator**: Now that we talked about fruits and vegetables for a bit, we are going to shift gears and talk about sports and activities that get you moving.

**Topic 2: Sports Activities (20 min)**

|  | **Question** | **Goal** |
| --- | --- | --- |
| 5. | Tell me about your thoughts on playing sports?  *Probes:*   - *When I first said “sports”, what was the first thought that came to your mind?* - *What sports do you like? What do you like about them?* - *Which do you not like? What is it that you don’t like about them?* - *Think about the most fun you’ve had playing a sport- whether a formal sport or just some fun activity where you’ve moved a little and got your heartbeat up. Tell me about that time.* | Identify child level attitudes on playing sports |
| 6. | What are some things that might motivate you all to play sports?  *Probes:*   - *Is there anything that could keep you physically active?* - *Do any of your friends play sports? Explain what you think motivates them to play.* | Identify child level motivation for playing sports. |
| 7. | What are your thoughts about playing sports with both boys and girls?  *Probes:*   - *How would you feel if you participated in a coed program?* - *How would you like it? How would you not like it?* | Identify child level perceptions of playing within a coed program |
| 8. | What sports or activities do kids play at your school?  *Probes:*   - *Do any of you play sports after school? What are your reasons for not playing sports after school?* - *What might make you want to get involved in sports after school?* - *What worries would you have about playing sports after school?* - *What would your family think about you playing sports after school?* - *What would your friends or other kids as school think about you playing sports after school?* | Identify child’s concerns about playing sports related to themselves and the family/peer level |
| 9. | If we were to start this program to get kids playing more sports at your school, what problems would we have?  *Probe:*   - *Can you describe any barriers to playing sports at your school?* | Identify school level barriers for implementation of program |

**Moderator**: Now we are going to do a quick activity.

*Use Easel Notepad to write down lists and recipes. Use the social cognitive theory model on notepad easel to identify facilitators for implementing the program. During the discussion, write down participants’ motivation, self-efficacy, experiences and interests under a personal level. Write down any type of social support and reinforcement opportunities the school or community has to support the program.*

**CBPR Activity: Social Ecological Model (30 min)**

|  | **Question** | **Goal** |
| --- | --- | --- |
| 10. | What are some sports activities you all would like to play?  *Probes:*   - *Explain why you all are most interested in these sports*   **Make a list on an easel notepad of all the sports activities they enjoy. Use this list later to probe on motivation and self-efficacy.*  **Probe with prepared examples of non-traditional sports that have been popular in similar contexts (e.g. Step, rugby, Zumba…)* | Identify sport interest from personal experience |
| 11. | If you were to play a (name specific sport), how would you think you’d play?  *Probes:*   - *How could we make you feel better about playing?* | Identify perceived self-efficacy about playing sports |
| 12. | Can you explain how the school could help generate sport interest for other middle school kids at your school?  *Probes:*   - *What else would the school have to do to make your ideas for new sport programs possible?* - *Tell me how your family can help you play the sports you like?*   **Write down participants' ideas under environmental level. Some of these ideas can be referred for recruitment/retention during intervention* | Identify sport interest as an opportunity to engage at an environmental level |
| 13. | Thinking about your favorite meal you drew, what are some fruits and vegetables you all would eat with the meal that you enjoy?  *Probe:*   - *Describe any fruits and vegetables you all would eat as a snack.*   **Have the participants rank the list of top fruits and vegetables they really like. You can use the ranking list as probes for identifying recipes they and their families would like to eat.* | Identify fruits and vegetables a child currently enjoys from personal experience. |
| 14. | Can you describe any meals with fruits and vegetables your family would enjoy?  **Write down recipes their family would like to eat. Rank the recipes from most tasty to not so tasty for the family. Use the top-ranking fruits and veggies they liked as probes to identify recipes.* | Identify recipes family would enjoy at an environmental level |

**UMKC YES Initiative**

Phase 1 - Parent Focus Group Guide

**Aims:**

Identify the target population’s preferred produce that may include dark leafy greens, red and orange vegetables and methods to reduce consumption of sugar-sweetened beverages among the target population. Identify barriers, concerns, and experiences accessing healthy foods.

Identify engaging sports activities for target population. Identify barriers, concerns, and experiences with performing activities.

**Time:** 60-90 minutes

**Supplies:**

- Audio recorders
- Notepad
- Easel notepads
- Markers
- Pencils
- Demographic survey
- Incentive
- Meal/Snacks
- Name tags

**Introduction:**

**Moderator**: Hi everyone, my name is Kimberly and this is *blank.* We are with Children’s Mercy and we work within the Health Services and Outcomes Research Division. We invited you here today asking for your help in starting a new before- or after- school program. We want to know your opinions and ideas about healthy eating in your family and your child playing sports.

The input you provide today will be used to help create this program and will remain confidential. We are audio recording the discussion today because we do not want to miss any of your comments. You all will say helpful things and we cannot write fast enough to get them all down. Our reports from the discussion will go back to Children’s Mercy and UMKC staff with no names attached and secured in our encrypted database.

Here is a document that talks about what it means to be in a study that we’ve given you. There is contact information for the study team as well as the hospital’s review board that oversees studies if you have any concerns or questions after today. Being in a study is completely voluntary, so you can choose to leave at any time or not answer any questions. By staying and participating in this discussion you are consenting to be in the study.

Do you have any questions about what it means to be in the study?

Let’s begin! Throughout the discussion today there will be a lot of ideas and some of you may be excited to talk about it. We encourage you to say anything that is on your mind. If you have a cell phone, please put it on silent mode.

**Topic 1: Fruits and Vegetables (20 min)**

|  | **Question** | **Goal** |
| --- | --- | --- |
|  | Icebreaker & Introductions: Guess Kids’ Favorite Meal  **Participants will be asked to introduce themselves on a first name basis. After, the moderator will ask parents to guess kids’ favorite meals from completed worksheets done at previous focus groups.* | Introductions, icebreaker |
| 1. | Thanks for playing along. Now I want you all to think about what is important to you when you decide what meals to cook for your family? Can you share your thoughts about what you think is most important?  *Probes:*   - *How do you decide what meals to cook for your family?* - *What makes a certain meal tasty in your family?* | Identify the deciding factors at the family level that affect the foods they eat. |
| 2. | What are your family’s thoughts about eating fruits and vegetables?  *Probes:*   - *What fruits or vegetables does your family like? What do you like about them?* - *Which ones do you not like? What is it that you don’t like about them?* - *What do your kids think of fruits or vegetables?* - *How often does your family eat fruits and vegetables at home?* | Identify family level attitudes and influences about eating fruits and vegetables. |
| 3. | What is it like for you to go grocery shopping in your community to get fruits and vegetables?  *Probes:*   - *Can you explain to me if there are enough stores around your community to buy fruits and vegetables?* - *What are your thoughts when shopping for fruits and vegetables?* | Identify family-level attitudes on access to fruits and vegetables |
| 4. | What makes it hard for your kids to eat fruits or vegetables?  *Probes:*   - *What makes it hard for your family to eat fruits and vegetables?* - *What makes it hard to eat fruits and vegetables if they are right in front of your child?* - *Can you explain any difficulties your child has eating fruits and vegetables at school?* - *Do you have any worries about fruits and vegetables?* - *Can you explain a time where your family had an obstacle or a barrier to eating fruits and vegetables?* | Identify barriers to eating fruits and vegetables at all levels |

**Moderator**: Now that we talked about fruits and vegetables for a bit, we are going to shift gears and talk about sports activities (fun physical activities, exercise activities) for your kids.

**Topic 2: Sports Activities (20 min)**

|  | **Question** | **Goal** |
| --- | --- | --- |
| 5. | What are your kids’ thoughts on playing sports?  *Probes:*   - *What sports does your child like?* - *Which sports do they not like?* - *Think about a time when your child was having a lot of fun playing a sport or just being active. Tell me how was it for them and for yourself?* - *What motivates your child to play sports?* - *What might make your child want to get involved in sports at school?* | Identify child level attitudes and motivation on playing sports from the parent perspective |
| 6. | How often does your family do sports or physical activities together?  *Probes:*   - *What sort of things do you do?* - *How does your child feel about these activities?* - *Do you and your child ever talk about their involvement in sports? What are these conversations like?* | Identify family level influences on child playing sports |
| 7. | Explain to me how you would feel if your child participated in a coed program?  *Probes:*   - *What are some thoughts you have about your child playing with boys and girls?* - *How might your child feel playing sports in a coed program?* | Identify parents perceptions of child playing in a coed program |
| 8. | What would be difficult for your family if your child was to play sports at school?  *Probes:*   - *What worries would you have about playing sports before- or after- school?* - *How might a before- or after- school program be designed to make these things easier?* - *What would your family say about your child playing sports?* | Identify parent’s concerns about child playing sports |
| 9. | If we were to start this program to get kids playing more sports at this school, what are some other problems would we have?  *Probe:*   - *Can you describe any barriers about your child playing sports at school?* | Identify school level barriers for implementation of program from parent perspective |

**Moderator**: Now we are going to do a quick activity. Before we start, I want to tell you all a little more about the program. The UMKC YES project will be for middle school students. The project’s goals will be to have a before- or after- school physical activity and nutrition program where we plan to provide opportunities for kids to be physically active and support healthy eating. By having the program before- or after- school, we can encourage the kids to play a sport or activity they enjoy to develop long-term physical activity habits. The activity today will help us learn what sports, fruits, and vegetables they might like so we can try to meet our goals. Let’s start the activity.

*Use Easel Notepad to write down lists and recipes. Use the social cognitive theory model on notepad easel to identify facilitators for implementing the program. During the discussion, write down participants’ motivation, self-efficacy, experiences and interests under a personal level from a parent's perspective. Write down any type of social support and reinforcement opportunities the school or community has to support the program.*

**CBPR Activity: Social Ecological Model (30 min)**

|  | **Question** | **Goal** |
| --- | --- | --- |
| 10. | What are some sports activities your child would like to play?  *Probes:*   - *What would make your child interested in playing a specific sport or activity?* - *What types of activities get your child moving at home?*   **Make a list on an easel notepad of all the sports activities parents believe their kids would enjoy. Use this list later to probe on motivation and self-efficacy.* | Identify child sport/activity interest from parental personal experience |
| 11. | Can you explain how the school could help generate sport interest for kids in school?  *Probes:*   - *What else would the school have to do to make your ideas for new sport program possible?* - *Tell me how your family can help your child play the sport or activity they like?*   **Write down parents’ ideas under environmental level. Some of these ideas can be referred for recruitment/retention during intervention* | Identify sports interest as an opportunity to engage at an environmental level from the parent perspective |
| 12. | What type of support could we provide to help your child play sports?  *Probes:*   - *What type of support could the school provide during the program for your child?* - *Can you tell me about any support families would need if their kids played sports?*   *Make a list of support parents may feel children need to participate in sports activities | Identify environmental support for family and child from parents’ perspectives |
| 13. | For this program, we plan to send home bags of fruits and vegetables from a local mobile market with the children. What are your thoughts on having this bag of healthy foods?  *Probes:*   - *How would the bag of fruits and vegetables be helpful to you at home?* - *What other items might be helpful for your family to eat healthy?* | Identify level of interest for a bag of fruits and vegetables program would hand how |
| 14. | Thinking about your favorite meal you described for your family, what are some fruits and vegetables you all would eat with the meal?  *Probe:*   - *Describe any fruits and vegetables your child would eat as a snack.* - *What are some fruits and vegetables you would enjoy?* - *What are some meals your family eats with fruits and vegetables?* - *Can you describe any meals with fruits and vegetables your family would enjoy?* | Identify fruits and vegetables child and family would enjoy with different recipes at an environmental level |

**UMKC YES Initiative**

Phase 1 - Parent Focus Group Guide

**Aims:**

Identify the target population’s preferred produce that may include dark leafy greens, red and orange vegetables and methods to reduce consumption of sugar-sweetened beverages among the target population. Identify barriers, concerns, and experiences accessing healthy foods.

Identify engaging sports activities for target population. Identify barriers, concerns, and experiences with performing activities.

**Time:** 60-90 minutes

**Supplies:**

- Audio recorders
- Notepad
- Easel notepads
- Markers
- Pencils
- Demographic survey
- Incentive
- Meal/Snacks
- Name tags

**Introduction:**

**Moderator**: Hi everyone, my name is Kimberly and this is *blank.* We are with Children’s Mercy and we work within the Health Services and Outcomes Research Division. We invited you here today asking for your help in starting a new before- or after- school program. We want to know your opinions and ideas about healthy eating in your family and your child playing sports.

The input you provide today will be used to help create this program and will remain confidential. We are audio recording the discussion today because we do not want to miss any of your comments. You all will say helpful things and we cannot write fast enough to get them all down. Our reports from the discussion will go back to Children’s Mercy and UMKC staff with no names attached and secured in our encrypted database.

Here is a document that talks about what it means to be in a study that we’ve given you. There is contact information for the study team as well as the hospital’s review board that oversees studies if you have any concerns or questions after today. Being in a study is completely voluntary, so you can choose to leave at any time or not answer any questions. By staying and participating in this discussion you are consenting to be in the study.

Do you have any questions about what it means to be in the study?

Let’s begin! Throughout the discussion today there will be a lot of ideas and some of you may be excited to talk about it. We encourage you to say anything that is on your mind. If you have a cell phone, please put it on silent mode.

**Topic 1: Fruits and Vegetables (20 min)**

|  | **Question** | **Goal** |
| --- | --- | --- |
|  | Icebreaker & Introductions: Guess Kids’ Favorite Meal  **Participants will be asked to introduce themselves on a first name basis. After, the moderator will ask parents to guess kids’ favorite meals from completed worksheets done at previous focus groups.* | Introductions, icebreaker |
| 1. | Thanks for playing along. Now I want you all to think about what is important to you when you decide what meals to cook for your family? Can you share your thoughts about what you think is most important?  *Probes:*   - *How do you decide what meals to cook for your family?* - *What makes a certain meal tasty in your family?* | Identify the deciding factors at the family level that affect the foods they eat. |
| 2. | What are your family’s thoughts about eating fruits and vegetables?  *Probes:*   - *What fruits or vegetables does your family like? What do you like about them?* - *Which ones do you not like? What is it that you don’t like about them?* - *What do your kids think of fruits or vegetables?* - *How often does your family eat fruits and vegetables at home?* | Identify family level attitudes and influences about eating fruits and vegetables. |
| 3. | What is it like for you to go grocery shopping in your community to get fruits and vegetables?  *Probes:*   - *Can you explain to me if there are enough stores around your community to buy fruits and vegetables?* - *What are your thoughts when shopping for fruits and vegetables?* | Identify family-level attitudes on access to fruits and vegetables |
| 4. | What makes it hard for your kids to eat fruits or vegetables?  *Probes:*   - *What makes it hard for your family to eat fruits and vegetables?* - *What makes it hard to eat fruits and vegetables if they are right in front of your child?* - *Can you explain any difficulties your child has eating fruits and vegetables at school?* - *Do you have any worries about fruits and vegetables?* - *Can you explain a time where your family had an obstacle or a barrier to eating fruits and vegetables?* | Identify barriers to eating fruits and vegetables at all levels |

**Moderator**: Now that we talked about fruits and vegetables for a bit, we are going to shift gears and talk about sports activities (fun physical activities, exercise activities) for your kids.

**Topic 2: Sports Activities (20 min)**

|  | **Question** | **Goal** |
| --- | --- | --- |
| 5. | What are your kids’ thoughts on playing sports?  *Probes:*   - *What sports does your child like?* - *Which sports do they not like?* - *Think about a time when your child was having a lot of fun playing a sport or just being active. Tell me how was it for them and for yourself?* - *What motivates your child to play sports?* - *What might make your child want to get involved in sports at school?* | Identify child level attitudes and motivation on playing sports from the parent perspective |
| 6. | How often does your family do sports or physical activities together?  *Probes:*   - *What sort of things do you do?* - *How does your child feel about these activities?* - *Do you and your child ever talk about their involvement in sports? What are these conversations like?* | Identify family level influences on child playing sports |
| 7. | Explain to me how you would feel if your child participated in a coed program?  *Probes:*   - *What are some thoughts you have about your child playing with boys and girls?* - *How might your child feel playing sports in a coed program?* | Identify parents perceptions of child playing in a coed program |
| 8. | What would be difficult for your family if your child was to play sports at school?  *Probes:*   - *What worries would you have about playing sports before- or after- school?* - *How might a before- or after- school program be designed to make these things easier?* - *What would your family say about your child playing sports?* | Identify parent’s concerns about child playing sports |
| 9. | If we were to start this program to get kids playing more sports at this school, what are some other problems would we have?  *Probe:*   - *Can you describe any barriers about your child playing sports at school?* | Identify school level barriers for implementation of program from parent perspective |

**Moderator**: Now we are going to do a quick activity. Before we start, I want to tell you all a little more about the program. The UMKC YES project will be for middle school students. The project’s goals will be to have a before- or after- school physical activity and nutrition program where we plan to provide opportunities for kids to be physically active and support healthy eating. By having the program before- or after- school, we can encourage the kids to play a sport or activity they enjoy to develop long-term physical activity habits. The activity today will help us learn what sports, fruits, and vegetables they might like so we can try to meet our goals. Let’s start the activity.

*Use Easel Notepad to write down lists and recipes. Use the social cognitive theory model on notepad easel to identify facilitators for implementing the program. During the discussion, write down participants’ motivation, self-efficacy, experiences and interests under a personal level from a parent's perspective. Write down any type of social support and reinforcement opportunities the school or community has to support the program.*

**CBPR Activity: Social Ecological Model (30 min)**

|  | **Question** | **Goal** |
| --- | --- | --- |
| 10. | What are some sports activities your child would like to play?  *Probes:*   - *What would make your child interested in playing a specific sport or activity?* - *What types of activities get your child moving at home?*   **Make a list on an easel notepad of all the sports activities parents believe their kids would enjoy. Use this list later to probe on motivation and self-efficacy.* | Identify child sport/activity interest from parental personal experience |
| 11. | Can you explain how the school could help generate sport interest for kids in school?  *Probes:*   - *What else would the school have to do to make your ideas for new sport program possible?* - *Tell me how your family can help your child play the sport or activity they like?*   **Write down parents’ ideas under environmental level. Some of these ideas can be referred for recruitment/retention during intervention* | Identify sports interest as an opportunity to engage at an environmental level from the parent perspective |
| 12. | What type of support could we provide to help your child play sports?  *Probes:*   - *What type of support could the school provide during the program for your child?* - *Can you tell me about any support families would need if their kids played sports?*   *Make a list of support parents may feel children need to participate in sports activities | Identify environmental support for family and child from parents’ perspectives |
| 13. | For this program, we plan to send home bags of fruits and vegetables from a local mobile market with the children. What are your thoughts on having this bag of healthy foods?  *Probes:*   - *How would the bag of fruits and vegetables be helpful to you at home?* - *What other items might be helpful for your family to eat healthy?* | Identify level of interest for a bag of fruits and vegetables program would hand how |
| 14. | Thinking about your favorite meal you described for your family, what are some fruits and vegetables you all would eat with the meal?  *Probe:*   - *Describe any fruits and vegetables your child would eat as a snack.* - *What are some fruits and vegetables you would enjoy?* - *What are some meals your family eats with fruits and vegetables?* - *Can you describe any meals with fruits and vegetables your family would enjoy?* | Identify fruits and vegetables child and family would enjoy with different recipes at an environmental level |

**UMKC YES Initiative**

## Phase 2 - Parent Focus Group Guide: COVID Adaptation

**Aims:**

Identify the target population’s preferred produce that may include dark leafy greens, red and orange vegetables and methods to reduce consumption of sugar-sweetened beverages among target population. Identify barriers, concerns, and experiences accessing healthy foods.

Identify engaging sports activities for target population. Identify barriers, concerns, and experiences with performing activities.

**Time:** 60-90 minutes

**Supplies:**

- Audio recorders
- Zoom login
- Attendance sheet (REDCap report)
- Stable internet
- Any smart device to access Teams or Zoom
- REDCap Link for Demographic survey
- Incentive (UMKC)

**Introduction:**

**Moderator**: Hi everyone, my name *(say your name)* and this is *(introduce others on study team).* We are with Children’s Mercy. We invited you here today asking for your thoughts and opinions about the UMKC YES (Youth Engagement in Sports) program you or your kids may started when you were in school. Since coronavirus started, we also want to learn how we can transition the program to being online if we need to. We also want to get your feedback about how we can help you learn more about nutrition and what favorite activities you like to play.

You should have all been sent a document that talks about what it means to be in the study. You also may have discussed it with one of our study members over the phone when you first signed up, but I’m going to post it in the chat box again so we can all go over it.

[Go over all consent points, emphasizing:]

- The input you provide today will be used to help create and improve this program and will remain confidential. We are going to record this discussion today because we do not want to miss any of your important comments. You all will say helpful things today and we cannot write fast enough to get them all down. The recording will not be video, it will just write down the words you say. Our reports from the discussion will go back to Children’s Mercy staff with no names attached and secured in our encrypted database.
- There is contact information for the study team as well as the hospital’s review board that oversees studies if you have any concerns or questions after today. Being in a study is completely voluntary, so you can choose to leave at any time or not answer any questions. By staying and participating in this discussion you are consenting to be in the study.
- Do you have any questions about what it means to be in the study?

*After everyone has logged on:*

Before we begin. I want to ask if you all can be in a quiet place or in an area with few distractions so we can all be engaged in our discussion today. Everyone’s opinions matter and it is important we can listen and chime in when we have something valuable to say.

Let’s begin! Throughout the discussion today there will be a lot of ideas and some of you may be excited to talk about it. We encourage you to say anything that is on your mind. This is really a conversation between you all- you can chime in and comment on someone’s ideas or thought. Feel free to speak directly or use the chat box if you prefer.

Before we start introducing ourselves, we are going to do a roll call of all the people who have signed up to participate. We ask that you say “HERE.” If you are having issues with your mic type “HERE” in the chat box if you are present. This will help us know who attended today’s discussion and who will receive an electronic gift card for today’s participation.

**Topic 1: General questions and in-person program activities**

|  | **Question** | **Goal** |
| --- | --- | --- |
|  | Icebreaker & Introductions: Kids’ Favorite Meal *[do NOT start until after attendance is taken]*  To start, we are going to go around and introduce yourself. You can say your first name or any name you would like to be called today. After you share who you are, as and icebreaker I would like you to describe your child’s favorite meal.  *Have participants share openly their first name or the name they wish to be called after row call. If participants mic does not work we should ask them to utilize the chat box.*  *(allow time before moving to the next item and to let the person in charge of the chat to read any responses.)* | Introductions, icebreaker |
| **Topic 1a: Fruits and vegetables** | | |
|  | Thanks for sharing! Now I want to know if you can tell me more about what are your family’s thoughts about eating fruits and vegetables?  *Probes:*   - *What do your kids think of fruits or vegetables?* - *What do other members of your family, like your kids’ grandparents, think of fruits and vegetables?* - *How often does your family eat fruits and vegetables at home?* | Identify family level attitudes and influences about eating fruits and vegetables. |
|  | What do you think can make it hard for your kids to eat enough fruits and vegetables?  *Probes:*   - *What makes it hard for your family to eat fruits and vegetables?* - *What makes it hard to eat fruits and vegetables if they are right in front of your child?* - *Can you explain any difficulties your child has eating fruits and vegetables at school?* - *Do you have any worries about fruits and vegetables?* | Identify barriers to eating fruits and vegetables at all levels |
|  | Thanks for sharing. Now I want you all to think about what is important to you when you decide about meals for your family? Can you share your thoughts about what is most important to you?  *Probes:*   - *How do you decide what meals to give your family?* - *What makes a certain meal tasty in your family?* - *Who is involved in cooking/preparing your meals at home?* | Identify the deciding factors at the family level that affect the foods they eat. |
|  | What are your thoughts about recipes?  *Probes:*   - *How often do you use recipes?* - *Where do you find recipes or ideas?*     What might get you excited about a new recipe?  What would make you want to learn or try a new recipe?  If the program provided recipes to you or your family to try, what is the best way to get those to you?  *Probes:*   - *social media (what platform- snapchat, fb, Pinterest, other), online search (google, youtube?) video , cookbook, email*   *Probes:*   - *What types of social media do you use the most?* - *If we wanted to share recipes with you, how could we get families to follow us on the social media?* | Identify modes of communicating recipes and cooking information to students and families |
|  | Since coronavirus happened, can you tell me what, if any, changes there been in the foods your family eats?  *Probes:*   - *Do you have any worries about the foods you eat?*   Have there been any changes in your grocery shopping?  *Probes:*   - *Have there been any changes in what it is like to get fruits and vegetables?* | Identify family influences on food during COVID  Identify family-level attitudes on access to fruits and vegetables |
| **Topic 1b: Sports** | | |
|  | Great! Now we are going to shift gears a bit and I want to ask you some questions about your thoughts on kids playing sports or activities.  First, let’s talk about what things were like when kids were going to school every day. During school, how did your kids’ feel about playing sports?  *Probes:*   - *What sports does your child like?* - *Which sports do they not like?* - *Think about a time when your child was having a lot of fun playing a sport or just being active. Tell me how was it for them and for yourself?* - *What motivates your child to play sports?* - *What might make your child want to get involved in sports or be active in the program?*   What makes it hard for your child to play sports or be active?  *Probe:*   - *What concerns do you have about your child doing sports or being active?* | Identify child level attitudes and motivation on playing sports from the parent perspective |
|  | How often does your family do sports or physical activities together?  *Probes:*   - *What sort of things do you do?* - *How does your child feel about these activities?* - *Do you and your child ever talk about their involvement in sports? What are these conversations like?* | Identify family level influences on child playing sports |
|  | For other focus groups, kids mentioned having goals could help them be active. Can you think of any goals your kids have now that helps them be active?  *Probe:*   - *If so, what was that like for them to have that goal or challenge?*   What do you think about doing a challenge like 10,000 steps or a certain number of minutes, as part of this afterschool program?  *Probes:*   - *What would your child like about that?* - *What would make it work for your child?* - *What would your child not like about doing that?* - *What would make it hard for your child to participate?* | Identify parent’s concerns about child playing sports |

**Topic 2: Online program adaptations**

|  | **Question** | **Goal** |
| --- | --- | --- |
|  | Now I’d like to get your opinions on the program if we had to start it up virtually.  First of all, think about the last few months of school, and what your child’s school did for online learning. What worked well that we might be able to do in this program?  *Probes:*   - *What didn’t work well that that we would want to avoid?*   If we were to continue the program while your kids are still at home, what are some ways they might like to learn about fruits and vegetables?  ***Example Probes:***   - *Take part in an online cooking lesson* - *Watch cooking videos/shows* - *Make cooking videos to share online* - *Search for healthy recipes together* - *Taste test different fruits or veggies/healthy recipes* - *Learn about how to pick out and store fruits and vegetables* - *Pick a recipe and try to make it “go viral”* - *Learn about the ways fruits and vegetables can help your health*   Can you explain to me any ideas you might think could engage your child in learning more about nutrition online?  *Probes:*   - *What are your thoughts on learning about fruits and vegetables online with your kids?* - *Are they already learning about fruits and vegetables in any way?* | Identify parent thoughts for delivering the nutrition education online |
|  | If the program were online, we would also have to engage kids in doing physical activity or sports at home. What are some activities or sports they play at home when they are not in school?  *Probes:*   - *What activities might they play at home?* - *Can you explain if your children prefer in-person or online activities?*   What are your thoughts on how we can motivate kids to be active at home?  *Probes:*   - *How can we keep them engaged online?* - *What might make it hard for them to play sports or activities at home?* - *What are some worries you might have about them being active at home?*   One of the things we’ve heard from other groups is that kids really like doing sports or activities with friends. If the program was online and not in-person with friends, how would do you see the kids having fun while staying social?   - *What would your kids think of joining “teams” with their friends to complete different physical activity challenges (like step goals)?* | Identify parents’ perspective on kids playing activities at home and their motivation. |
|  | How likely would your child participate in the program if it was online?   - *What challenges do you foresee that we might face?* - *What worries do you have about the program being online?* - *What is internet access like at home?* - *What is your child’s access to technology at home?* | Identify parents’ perspective on child willingness to participate online and challenges the program may face |

**Topic 3: Changing the name of the program**

|  | What do you all think of the name YES (Youth Engagement Sports) for this program?  *Probe: We have heard from some participants that the name of this program, YES (youth engagement in sports) gives the impression that this is a competitive sports program. This program is not meant to be competitive for you all. It is meant to be a program that helps you engage more in physical activity.*  If you could change the name of this program, what would you name it. You can share or write your ideas in the chatbox!  *Probe:*   - *What type of names would your kids like?*   Can you explain what makes these names better for the program? | Identify new name for program from parents |
| --- | --- | --- |

**La iniciativa YES de la universidad de Missouri-Kansas City (UMKC por sus siglas en inglés)**

**Guía para el grupo de enfoque de padres: adaptación coronavirus**

**Objetivos:**

Identificar los productos favoritos de la población designada. Los productos pueden incluir vegetales de hojas verdes oscuras, vegetales de colores rojo y naranja. También, maneras de reducir el consumo de bebidas azucaradas entre la población designada. Identificar barreras, preocupaciones y experiencias para encontrar alimentos saludables.

Identificar actividades deportivas interesantes para la población designada. Identificar barreras, preocupaciones, y experiencias con estas actividades.

**Duración**: 60-90 minutos

**Materiales para la reunión:**

- Grabadoras

- Información para entrar a la plataforma de Zoom (como nombre de usuario y clave)

- Hoja de asistencia

- Conexión de internet estable

- Cualquier tipo de dispositivo inteligente electrónico para acceder a Zoom.

- Enlace de REDCap para la encuesta demográfica

- Incentivos (UMKC)

**introducción:**

**Presentador:** Hola a todos, mi nombre es (digo mi nombre e introducir a los demás del grupo). Nosotros trabajamos para el hospital de Children’s Mercy. Los hemos invitado el día de hoy a que conversen con nosotros porque queremos saber sus opiniones acerca del programa de YES (que en español significa: Participación de jóvenes en deportes) creado por la universidad de Missouri-Kansas City (UMKC por sus siglas en ingles) en el cual sus hijos empezaron a participar cuando aún estaban en la escuela. Ya que el coronavirus empezó, también queremos aprender cómo podemos cambiar el programa a que este se transmita en línea en caso de que sea necesario. También, queremos que nos den su opinión de cómo podemos ayudar a sus hijos a aprender más sobre nutrición y cuáles cuales son las actividades físicas/deportes favoritos de sus hijos.

Todos debieron haber recibido un documento que habla sobre lo que significa estar en este estudio. Puede que hayan hablado con uno de los miembros del estudio por teléfono cuando recién se inscribieron para participar en el estudio, pero de todos modos pondré ese documento nuevamente en el chat para que lo revisemos juntos.

Mencionar todos los puntos de consentimiento, enfocarse en:

Las aportaciones que nos den el día de hoy serán usadas para crear y mejorar el programa de YES y todo lo que digan el día de hoy será confidencial. Vamos a grabar el audio de la conversación de hoy porque no queremos perder ninguno de sus comentarios. Todos dirán cosas importantes que nos ayudarán. Lamentablemente no podemos escribir tan rápido como nos gustaría para poder escribir todo.

- La grabación no será video, solamente será audio y grabaremos las palabras que digan. El reporte de la conversación del día de hoy regresará al hospital de Children’s Mercy, pero no tendrá ninguno de sus nombres y lo aseguraremos en una base de datos que solo puede ser accedida por medio de un código secreto que solo sabemos los miembros del estudio.
- Hay información disponible para contactar al personal del estudio, también como del instituto de revisiones del hospital quien se encarga del estudio, por si ustedes tienen alguna preocupación o pregunta después de hoy. Estar en este estudio es completamente voluntario, entonces usted puede retirarse en cualquier momento o no contestar a ninguna pregunta. Al quedarse están dando su consentimiento a participar en el estudio.
- ¿Tienen alguna pregunta acerca de lo que significa estar en el estudio?

después de que todos se hayan conectado:

Antes de que comencemos a platicar, me gustaría pedirles que por favor se mantengan en un lugar silencioso o en un lugar con pocas distracciones para que todos podamos participar en la discusión de hoy. Las opiniones de todos son importantes y es muy importante que escuchemos y que guardemos silencio cuando alguien tenga algo que decir.

¡Empecemos! Durante la conversación del día de hoy habrá muchas ideas y muchos de ustedes tal vez se emocionen al hablar de ellas. Le pedimos que diga lo que le venga a la mente. En realidad, esta es una conversación entre todos y siéntanse con la libertad de contestar a los comentarios de los demás. Siéntase libre de hablar directamente o de usar el chat si lo prefiere.

Antes que empecemos a introducirnos, voy a llamar asistencia de todas las personas quienes se inscribieron para participar. Le pedimos que por favor diga “aquí”. Si tiene algún problema con su micrófono por favor escriba en el chat “Aquí” para indicar que está presente. Esto nos ayudara a saber quien asistió a la discusión de hoy y quien recibirá su tarjeta electrónica de regalo.

Decirles a los padres que se cambien el nombre. (compartir un video de cómo hacerlo)

**Tema 1: Preguntas generales y actividad del programa en persona**

| Preguntas | Objetivos |
| --- | --- |
| *Vamos a romper el hielo y a introducirnos: la comida favorita de su hijo/a. [no empezar antes de tomar asistencia]*  Para empezar, vamos a introducirnos. Usted puede decir su primer nombre o cualquier nombre que guste que le llamemos el día de hoy. Después que diga quien es, para romper el hielo me gustaría que dijera la comida favorita de su hijo/a o hijos.  *Permitir que los participantes abiertamente compartan su primer nombre o el que deseen que los llamen después de tomar asistencia. Si los micrófonos de los participantes no funcionan debemos pedirles que usen el chat.*  *(permitir cierto tiempo antes de continuar con el siguiente tema y permitir a la persona encargada del chat leer cualquier respuesta)* | Introducción y rompe hielos. |
| **Tema 1A: Frutas y Vegetales** |  |
| ¡Gracias por compartir! Ahora queremos saber si nos pueden decir; ¿qué piensa su familia acerca de comer frutas y vegetales?  Preguntas para indagar más:   - Por ejemplo, ¿qué piensan sus hijos acerca de las frutas y vegetales? - ¿Qué tal otros miembros de su familia, como los abuelos de sus hijos, que piensan ellos de las frutas y vegetales? - ¿Qué tan seguido come frutas y vegetales en casa? | Identificar actitudes e influencias sobre comer frutas y vegetales a nivel familiar. |
| ¿Hay algo que hace difícil que sus hijos coman suficientes frutas y vegetales?  Preguntas para indagar más:   - ¿Qué tal su familia en general, hay algo que hace difícil que usted y su familia coman frutas y vegetales? - ¿Que hace difícil que sus hijos coman frutas y vegetales incluso cuando los tienen enfrente? - ¿Puede mencionar algunos problemas que su hijo tiene al comer frutas y vegetales en la escuela? - ¿Ustedes tienen alguna preocupación acerca de las frutas y vegetales? | Identificar barreras al comer frutas y vegetales en todos los niveles |
| Gracias por compartir, ahora quiero que piensen, ¿qué es lo más importante para ustedes cuando están pensando en que cocinar para su familia?  Preguntas para indagar más:   - ¿Como deciden que darles de comer a su familia? - ¿Hay algo en especial que haga la comida más rica? - Quien se encarga de cocinar/ prepara los alimentes en casa? | Identificar factores de decisión a nivel familiar que afectan las comidas que comen |
| ¿Qué piensan sobre las recenas de cocina?   - ¿Ustedes usan recetas para cocinar? - ¿Cada cuando usan ustedes recetas para cocinar? - ¿Dónde encuentras ideas o recetas para cocinar?   ¿Que podría hacer que ustedes quieran tratar una recena nueva?  En el pasado, le dimos a los estudiantes una bolsa con frutas y vegetales y una receta para cocinar. ¿Qué piensan sobre eso?  Preguntas para indagar más:   - ¿Me pueden decir si usaría estas recetas? - ¿Cómo podríamos facilitarle el uso de las recetas? | Identificar modos para cocinar a los estudiantes y familiares recetas y información de como cocinar |
| ¿Desde que empezó el coronavirus, me pueden decir si ha habido algún cambio en la alimentación de su familia?  Preguntas para indagar más:   - ¿tal vez alguna tiene alguna preocupación nueva acerca de los alimentos que comen?   ¿Ha habido algunos cambios en sus compras de alimentos?  Preguntas para indagar más:   - ¿Ha habido algunos cambios en conseguir frutas y vegetales? | Identificar que influye los alimentos durante el coronavirus  Identificar actitudes sobre el acceso de frutas y vegetales a nivel familiar |
| **Tema 1B: Deportes o actividad física** | |
| ¡Genial! Ahora vamos a cambiar un poco la dirección de esta platica y quiero preguntarles unas preguntas sobre lo que piensan ustedes de que sus hijos jueguen algún deporte o hagan alguna actividad física.  Primero, hablemos sobre cómo eran las cosas cuando sus hijos iban a la escuela todos los días. ¿Durante la escuela, como se sentían sus hijos al jugar deportes?  Preguntas para indagar más:   - ¿Qué deportes les gustan a sus hijos? - ¿Qué deportes no les gustan? - Piensen en algún momento donde su hijo/a se estaba divirtiendo bastante jugando un deporte o alguna actividad física. díganme como fue esa experiencia para ellos y para ustedes? - ¿Qué es lo que motiva a sus hijos a jugar deportes? - Que cosas pueden inspirar a sus hijo/a a participar en deportes o participar en actividades físicas que el programa pueda dar?   ¿Hay algo que no le permita a su hijo participar en deportes o actividades físicas?  Preguntas para indagar más:   - ¿Qué preocupaciones tiene usted cuando su hijo practica algún deporte o se mantiene activo? | Identificar actitudes y motivadores hacia los deportes desde la perspectiva de los padres en el nivel de los niños |
| ¿Qué tan seguido su familia practica algún deporte o actividad física juntos?  Preguntas para indagar más:   - ¿Qué tipo de cosas hacen? - ¿Como se sienten sus hijos al hacer esas actividades? - ¿Usted y su hijo hablan sobre la participación de ellos en deportes? ¿Como son esas conversaciones? | Identificar influencias de los niños al jugar deportes a nivel familiar |
| En otros grupos, unos jóvenes mencionaron que tener metas les podría ayudar a mantenerse activos. ¿Pueden pensar en alguna meta que sus hijos tengan ahora que les ayuda a mantenerse activos?  Preguntas para indagar más:   - Si hay algo, como fue eso para ellos, ¿tener una meta o un objetivo?   ¿Qué piensan sobre hacer un desafío de 10,000 pasos o estar activo por cierto tiempo, como parte de un programa después de la escuela?  Preguntas para indagar más:   - ¿Qué les gustaría a sus hijos sobre esa idea? - ¿Qué haría que sus hijos participaran en ese desafío? - ¿Que no les gustaría a sus hijos sobre esa idea? | Identificar las preocupaciones de los padres cuando sus hijos juegan deportes |

**Tema 2: adaptaciones para el programa en línea**

| Preguntas | Objetivos |
| --- | --- |
| Si el programa de YES continuara virtualmente/en línea mientras sus hijos están en casa, pueden pensar en algunas maneras de cómo les gustaría a sus hijos aprender sobre frutas y vegetales?  Preguntas para indagar más:   - Participar en una clase de cocina por internet - Ver videos o programas de cocina - Hacer un video de cocina y compartirlo por internet - Buscar recetas saludables juntos - Probar diferentes frutas y vegetables/recetas saludables - Escoger una receta y tratar de que se haga viral en las redes sociales. - Aprender más sobre como las frutas y vegetales pueden ayudar a mantenerse saludable   Pueden pensar en alguna idea que pueda animar a sus hijos a participar en el aprendizaje de nutrición de una manera virtual o por internet.  Preguntas para indagar más:   - ¿Qué piensan ustedes sobre el aprendizaje de sus hijos sobre las frutas y vegetales por medio de internet? - ¿Hay algo que ellos ya estén aprendiendo sobre las frutas y vegetales?   Pensando en los últimos meses de escuela virtual/en línea que han tenido sus hijos, y lo que sus hijos han tenido que hacer para aprender en línea, ¿qué ha funcionado bien y que tal vez nosotros podríamos usar para este programa?  Ahora, pueden pensar en algo que hizo difícil el aprendizaje virtual/o por internet? ¿Algo que tal vez debamos evitar? | Identificar las opiniones de los padres acerca de difundir educación sobre nutrición virtualmente |
| También, hay una posibilidad que la parte de actividad física tuviera que ser virtualmente. Tendríamos que tratar de que los jóvenes participen en actividad físicas o deportes desde casa. ¿Cuáles son algunas actividades físicas o deportes de que sus hijos juegan en casa cuando no están yendo a la escuela?  Preguntas para indagar más:   - ¿Hay algunos juegos que hagan en casa? - ¿Que prefieren sus hijos, actividades en persona o en línea?   ¿Qué piensan ustedes, como podemos motivar a sus hijos a mantenerse activos en casa?  Preguntas para indagar más:   - ¿Como podemos mantenerlos entretenidos virtualmente? - ¿Qué puede complicar que ellos hagan deportes o se mantengan activos desde casa? - ¿Ustedes, tienen alguna preocupación de que ellos se mantengan activos desde casa?   Una de las cosas que hemos oído de otros grupos que hemos entrevistado, es que los jóvenes les encanta hacer actividades con sus amigos. ¿Si el programa fuera virtual y no en persona con sus amigos, como visualizan ustedes a sus hijos divirtiéndose, pero también tratando de mantener un contacto virtual con otros jóvenes?  Como creen ustedes que sus hijos se sientan si pudieran conectase en una plataforma como Zoom (esta) con sus amigos y hacer diferentes actividades físicas o retos (como el de los 10 mil pasos) | Identificar las opiniones de los padres acerca de que sus hijos juegues en casa y las motivaciones de ellos |
| Que tan probable es que su hijo/a quiere participar si el programa fuera en línea?  ¿Qué complicaciones pueden ustedes anticipar?  ¿Qué preocupaciones tienen ustedes si el programa fuera en línea?  ¿Tienen acceso a internet en casa?  ¿Qué tanto acceso tecnológico tiene ustedes en casa? Como, por ejemplo, ¿celular, computadoras, iPad? Etc.? | Identificar las perspectivas de los padres acerca de posibles retos que se puedan encontrar conforme a la voluntad de sus hijos al participar virtualmente |

**Tema 3: Cambiando el nombre del programa**

| Preguntas | Objetivos |
| --- | --- |
| ¿Yo no sé si saben, pero el nombre del programa es “YES (por sus siglas en inglés)” es una abreviación para “Participación de jóvenes en deportes”? ¿Qué piensan ustedes sobre el nombre del programa?  Para indagar más: hemos oído de algunos participantes de que el nombre del programa les dé la impresión de que es un programa de deportes competitivos. Este programa no se trata de eso, lo que en realidad queremos dar a entender es que es un programa para ayudar a los jóvenes a mantenerse activos.  ¿Si ustedes pudieran cambian el nombre del programa, como le pondrían? Pueden decir sus ideas o escribirlas en el chat.  Preguntas para indagar más:  - ¿Piensen en algún nombre que a sus hijos les gustaría?  - ¿Porque escogieron ese nombre? | Identificar nuevos nombres para el programa desde el punto de vista de los padres |
